# Supplementary figures and images for: Human Antibody Domains and Fragments Targeting Neutrophil Elastase as Candidate Therapeutics for Cancer and Inflammation-Related Diseases
Source: Int J Mol Sci. 2021 Oct 15;22(20):11136. doi: 10.3390/ijms222011136 (PMC8539514; doi:10.3390/ijms222011136)

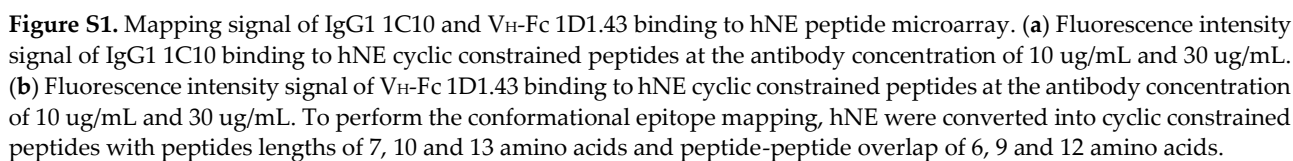

Supplement: Supplementary file 1 [file ijms-22-11136-s001.zip › ijms-1422536-supplementary.pdf]
